# Challenges and Open Issues in Transcatheter Mitral Valve Implantation: Smooth Seas Do Not Make Skillful Sailors

**DOI:** 10.3389/fcvm.2021.738756

**Published:** 2022-02-09

**Authors:** Giulio Russo, Francesco Maisano, Gianluca Massaro, Giuseppe Terlizzese, Enrica Mariano, Michela Bonanni, Andrea Matteucci, Andrea Bezzeccheri, Daniela Benedetto, Gaetano Chiricolo, Eugenio Martuscelli, Giuseppe Massimo Sangiorgi

**Affiliations:** ^1^Department of Biomedicine and Prevention, Policlinico Tor Vergata, Rome, Italy; ^2^Dipartimento di Scienze Cardiovascolari, Università Cattolica del Sacro Cuore, Rome, Italy; ^3^Cardio-Thoracic-Vascular Department, San Raffaele Institute, Milan, Italy

**Keywords:** transcatheter mitral valve repair, transcatheter mitral valve implantation, mitral regurgitation, valve-in-valve, valve-in-ring, valve-in-MAC

## Abstract

According to the European and American guidelines, surgery represents the treatment of choice for mitral valve (MV) disease. However, a number of patients are deemed unsuitable for surgery due to a prohibitive/high operative risk. In such cases, transcatheter therapies aiming at MV repair have been proven to be a valuable alternative and have been recently introduced in the latest American guidelines on valvular heart disease. Indeed, percutaneous repair techniques, particularly transcatheter edge-to-edge, have gained a broad experience and demonstrated to be safe and effective. However, given the complexity and heterogeneity of MV anatomy and pathology, transcatheter MV implantation (TMVI) has grown as a possible alternative to percutaneous MV repair. Current data about TMVI are still limited and come from different settings: valve-in-native MV, valve-in-valve (ViV), valve-in-ring (ViR), and valve-in-mitral annular calcification. Preliminary data are promising although several open issues still need to be addressed. This paper provides a comprehensive review of the available devices in the different clinical settings, to discuss potentialities, limitations, and future directions for TMVI.

## Introduction

Mitral regurgitation (MR) represents the most prevalent heart valve disease, and it is associated with a poor prognosis if left untreated (1). Currently, open-heart surgery is the gold standard treatment for severe MR. However, almost half of severe MR cases are unsuitable candidates for surgery due to a high operative risk (2). Consequently, transcatheter mitral valve (MV) therapies have developed over the last few years. While transcatheter MV repair techniques, especially edge-to-edge technology, are widely spread and a huge experience has been gained, over the last few years transcatheter MV replacement has been deeply studied as a promising alternative treatment. Several surgical procedures have inspired transcatheter devices to treat MR. Currently, there are multiple transcatheter strategies to treat MR: edge-to-edge clip repair, indirect and direct annuloplasty, chordal replacement, and MV replacement.

Nowadays, the MitraClip (Abbott Vascular, Santa Clara, CA, USA) represents the most widespread option for patients with symptomatic MR and a prohibitive surgical risk. Clinical studies have demonstrated its safety and efficacy, consequently, a class IIa recommendation has been included in the latest guidelines of both the American College of Cardiology/American Heart Association and the European Society of Cardiology (3, 4). Alongside the MitraClip, recently, the PASCAL (Edwards Lifesciences, Irvine, CA, USA) has further enriched the edge-to-edge technology.

However, MV anatomy, MR etiology, proper timing selection, and operators' experience might limit the field of application for transcatheter edge-to-edge repair (TEER) (5, 6). In this perspective, transcatheter MV implantation (TMVI) might widen the MV therapy toolbox by playing a complementary role with TEER.

In contrast to transcatheter aortic valve replacement (TAVR), transcatheter implantation of prosthetic valves in the mitral position remains to be experienced at an early stage. It is mainly based on balloon-expandable prosthetic valves, originally not designed for mitral position, in the setting of failed surgical bioprosthesis or rings (valve-in-valve, ViV or valve-in-ring, ViR) or in patients with severe mitral annular calcification (MAC) deemed at a high risk for surgery. Data about TMVI in native MV are encouraging despite being still limited.

The aim of this paper is to provide a comprehensive review of the available TMVI technologies to discuss their possible application in the different settings of MV disease with a glimpse of future directions.

## TMVI Outcomes

### ViV, ViR, and Valve-in-MAC

Almost one-third of patients undergoing MV surgery, either replacement or repair, need reoperation over a 10-year follow-up (7). In such cases, redo surgery mortality may reach up to 12%, dependent on patients' comorbidities (8, 9). Starting from these premises, the role of TMVI in the setting of ViV, ViR, and valve-in-MAC (ViMAC) has gained attention and has shown promising results.

The outcomes of TMVI for patients with degenerated bioprosthesis, failed annuloplasty rings, and severe MACs have been recently reported in a multicenter registry by Yoon et al. involving 521 patients (10). The enrolled population was represented by high-risk patients with the Society of Thoracic Surgeons' (STSs') mean score of 9%. Analyzing procedural and 30-day outcomes, an important finding was the heterogeneity of clinical results depending on the valve subgroup: ViV showed a high technical success (94%) with a 30-day and 1-year mortality of 6.2% and 14%, respectively. On the contrary, ViR and ViMAC represented the two different settings posing procedural challenges, with less encouraging results: 30-day mortality in ViR was 9.9%, and 1-year mortality in ViR was 30.6%. However, it is noteworthy that no specific data about the type of implanted ring were provided although ViR outcomes could be significantly influenced by ring type. Thus, no general conclusion could be drawn. The most complex scenario was represented by ViMAC, with a technical success of 62% while 30-day mortality and 1-year mortality were 34.5 and 62.8%, respectively.

The greatest concern emerging from this observational registry was about the risk left ventricle outflow tract obstruction (LVOTO): in the ViMAC, it was particularly high (39.7%), while it occurred less frequently in the ViV (2%) and the ViR (5%) group. Reinterventions, mostly represented by the residual atrial septal defect closure and by alcohol septal ablation (ASA), were needed in 10.9, 17.7, and in 22.4% of ViV, ViR, and ViMAC patients, respectively. MR degree at 30 days was almost completely abolished with only 6.6% of patients showing a moderate or higher MR degree.

In addition to this registry, the data obtained from the STS/Transcatheter Valve Therapies (STS/TVT) registry confirmed the same trend for the three groups (11). In ViR patients, a sub-analysis on the different types of rings (complete vs. incomplete and rigid vs. non-rigid) showed no significant differences between them although the sample size was small. Mortality was lower in the incomplete ring (4.7%) vs. complete ring (10.3%) subgroup. As a general statement, while rigid rings might be limited by the risk of paravalvular leak (PVL) and by prosthetic valve underexpansion, nonrigid rings may increase the risk for valve embolization. Of note, in the STS/TVT registry, the occurrence of LVOTO in the ViMAC group was much lower (10%) as compared to the abovementioned experience by Yoon (39.7%). The latter might be explained by the non-univocal definition used for LVOTO among these registries. In particular, the definition of LVOTO as an increase of mean gradient by 10 mmHg from baseline in the registry by Yoon was based on the Mitral Valve Academic Research Consortium (MVARC). Conversely, in the registry by Guerrero and coauthors, no standard definition was adopted among the different enrolling sites and some centers might have considered LVOTO as only those with hemodynamic compromise. In addition, a more careful screening process might have influenced the lower incidence of LVOT obstruction. Consequently, the overall burden was lower (2.3%) in LVOTO than in the Yoon registry (7.1%). The most common procedural complications were represented by the need for a second valve (3.7%) and by vascular access repair (3.3%). Echocardiographic findings were similar to the Yoon registry confirming that TMVI successfully abolishes MR in most patients. In particular, 3.3% of patients in the overall population showed MR ≥ 2+ at a 30-day follow-up with the ViV being the group with the best performance, and the ViR and ViMAC reporting 9.3 and 5.7% of MR ≥ 2+ at a 30-day follow-up, respectively. Main TMVI outcomes in the settings of ViV, ViR, and ViMAC are summarized in Table 1.

**Table 1:** Main clinical outcomes for valve-in-valve (ViV), valve-in-ring (ViR), and valve-in-mitral annular calcification (ViMAC).

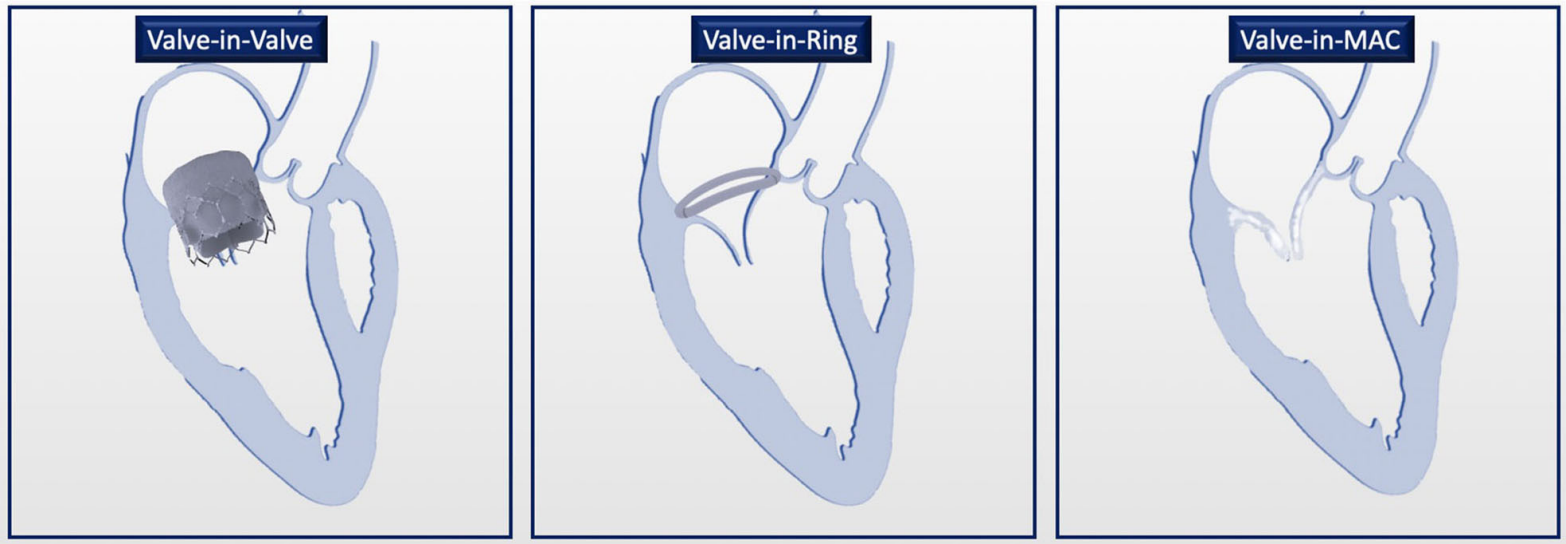						
	**Guerrero et al. (11)**	**Yoon et al. (10)**	**Guerrero et al. (11)**	**Yoon et al. (10)**	**Guerrero et al. (11)**	**Yoon et al. (10)**
	***N*** **= 680**	***N*** **= 322**	***N*** **= 680**	***N*** **= 322**	***N*** **= 680**	***N*** **= 322**
Procedural success	90.9%	73.6%	82.9%	57.4%	74%	41.4%
LVOT obstruction	0.7%	2.2%	4.9%	5.0%	10%	39.7%
Conversion to surgery	1.3%	0.9%	2.4%	2.8%	2.0%	8.6%
Need for a 2nd valve	1.5%	2.5%	7.3%	12.1%	14%	5.2%
Valve embolization	0.1%	0.9%	2.4%	1.4%	3%	6.9%
Residual MR ≥ 2	2.5%	5.6%	10.6%	18.4%	7.0%	13.8%
30-day MR ≥ 2	1.9%	3.3%	9.3%	12.6%	5.7%	13.2%
30-day mortality	8.1%	6.2%	11.5%	9.9%	21.8%	34.5%

*LVOT, left ventricle outflow tract; MR, mitral regurgitation*.

Recently, data focusing on transseptal TMVI in these three settings have been collected in the MITRAL trial (12): of the 30 patients enrolled in the ViV group, the procedural success was 100% and only one death was observed at both 30-day and 1-year follow-ups. In addition, most patients experienced an improvement in symptoms and all patients showed MR grade ≤ 1 after 1 year. Outcomes in the 30 ViR patients were less promising with a 66.7% technical success mainly due to the requirement of a second valve implantation in 6 patients. Mortality values were 6.7% and 23.3 at 30 days and 1 year, respectively.

The MITRAL trial also confirmed the worst results in the ViMAC group as compared to the other two groups (13): 30-day mortality was 16.7% reaching up to 34.5% after 1 year. LVOTO occurred in almost 10% of the cases, and preemptive ASA was performed in patients deemed at a high risk for LVOTO. However, in line with ViV and ViR, an improvement in symptoms and MR grade was observed among the survivors.

Based on the current available data, ViV might represent the first-line therapy for those with failing bioprosthesis, while ViR and ViMAC represent the two high-risk categories and further data are needed to evaluate the true safety and efficacy in these patients' subgroups.

### Valve-in-Native MV

Although several devices have been tested over the last few years, the experience gained in the field of native MV is still limited due to the high selection failure (14). Some anatomical criteria have been proposed to detect eligible candidates for TMVI based on CT scan characteristics (15): mitral annulus area > 8.6 cm^2^, mean mitral annulus systolic diameter ≤ 38.3 mm, aorto-mitral angulation > 130°, and the annulus-to-apex distance < 100 mm have shown to predict TMVI eligibility with a positive predictive value of 75% and a negative predictive value of 85.5%.

Currently, Tendyne (Abbott Vascular, Santa Clara, CA, USA) has received the CE approval and represents the device with the widest experience in the setting of native MV. Current available data for the most active TMVI devices in the setting of native MV are presented in Table 2.

**Table 2:** Main characteristics and clinical outcomes for transcatheter mitral valve implantation (TMVI) in native mitral valve. Mortality is meant at the longest follow-up.

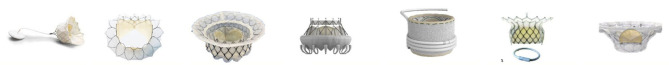							
	**Tendyne**	**Tiara**	**Intrepid**	**Evoque**	**Sapien**	**Highlife**	**Cardioval ve**
	**(Abbott)** ***N*** **= 100**	**(Neovasc)** ***N*** **= 79**	**(Medtronic)** ***N*** **= 50**	**(Edwards)** ***N*** **= 15**	**M3** **(Edwards)** ***N*** **= 15**	**(Highlife SAS**) ***N*** **= 15**	**(Valtech)** ***N*** **= 5**
**Device characteristics**							
Frame	Nitinol double frame SE	Nitinol SE	Nitinol double frame SE	Nitinol SE	Cobalt-Chromium SE	Nitinol SE	Nitinol SE
Leaflets	3 porcine	3 bovine	3 bovine	3 bovine	3 bovine	3 bovine	3 bovine
Anchoring mechanism	Apical Theter	Leaflet engagement	Small cleat + radial force	Annulus clamping	Nitinol dock system	External anchor	Leaflet grasping
Approach	Transapical	Transapical	Transapical	Transfemoral	Transfemoral	Transapical	Transfemoral
Delivery system, Fr	36	32-36	35	30	20	39	28
**Outcomes**							
FMR etiology, %	89	62	72	27	/	73	100
Technical success, %	97	93	98	93	87	73	100
Follow-up, days	416	30	173	30	30	30	30
Mortality, %	26	12	22	7	2	21	60

*E, balloon-expandable; FMR, functional mitral regurgitation; SE, self-expandable*.

## TMVI Devices

Several devices have been used for TMVI so far. Although they share many similarities, each device has its own peculiarities and, in particular, each has developed a different fixation and anchoring mechanism. Current data are too limited to establish which technology might be more promising in the future. The only Conformitè Européenne (CE) approved device is represented by Tendyne, whereas other devices collect data and are currently at the different stages of clinical study.

### Tendyne

**Figure 1:**
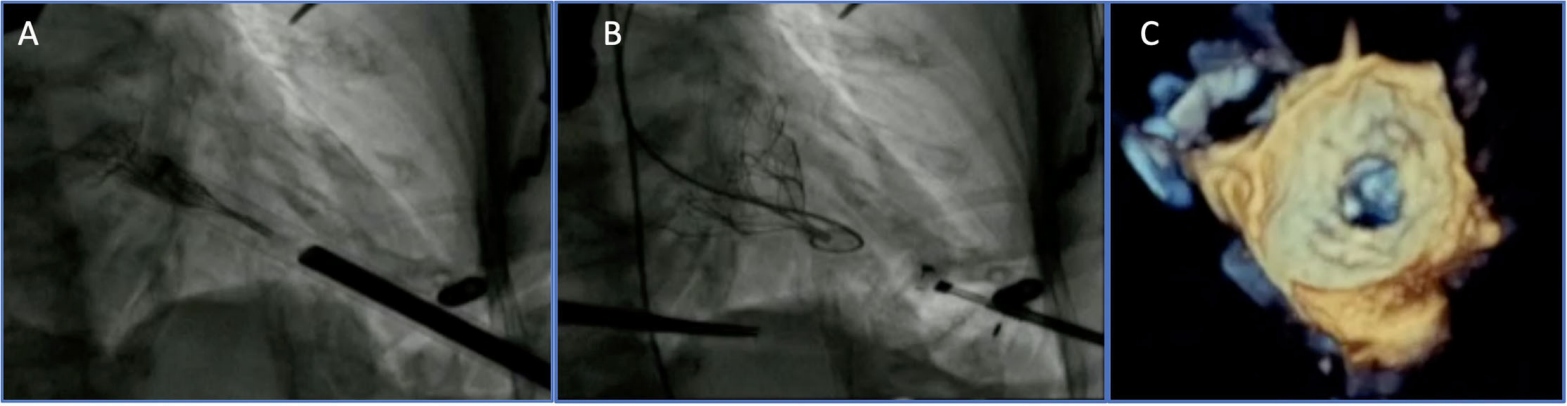
Transapical transcatheter mitral valve implantation (TMVI): Tendyne device before **(A)** and after implantation **(B,C)**.

The Tendyne valve (Abbott Vascular, Santa Clara, CA, USA) is a trileaflet porcine pericardial valve with a circular inner stent sutured to an outer nitinol stent. The outer stent is formed into a D-shaped body, which is designed to facilitate the sealing of the valve. It is implanted transapically and fixed through an apical pad that is tethered to the valve and, in this way, contributes to the apical closure (Figure 1). Of note, it is fully repositionable and retrievable.

It is the only CE approved device and the one with the widest available data to date. The Global Feasibility Study included 100 high-risk patients, mostly affected by secondary functional MR (89%) (16). Device implantation was successful in 97% of the cases while three implants were abandoned or retrieved. After 1 year, mortality was 26% and nine device-related adverse events were reported (three hemolysis and three thrombosis). More than 90% of patients showed none/grade I residual MR with a significant improvement in symptoms and quality of life. Such data were also confirmed after a 2-year follow-up with a 39% overall mortality rate and 93% of patients with none/trace residual MR (17). The valve has been also successfully used in the setting of ViMAC (18). Currently, the TENDER registry collects the data from European centers with central core-lab analyses and will provide a real-world perspective on the experience of Tendyne.

Moreover, the SUMMIT trial (NCT03433274) is randomized to Tendyne or to MitraClip with an estimated study completion date by 2026.

### Tiara

Tiara (Neovasc, Inc., Richmond, BC, Canada) consists of a trileaflet bovine pericardial valve mounted within a self-expanding nitinol alloy frame. Like Tendyne, it has a “D” shape to conform to the mitral annulus to reduce the risk of PVL and LVOTO. In addition, the atrial skirt provides further sealing against PVL, while the fixation mechanism, based on the combination of radial force and the three anchors capturing the native leaflets, also reduce the risk for LVOTO. The valve is implanted transapically although a transseptal system is under development.

The data of 79 patients obtained from the TIARA I, TIARA II, and the compassionate use showed successful implantations in 92% of the cases with no procedural deaths (19). After a 30-day follow-up, seven deaths were observed (compassionate procedures excluded). Performances of prostheses were excellent after a 1-year follow-up with all patients showing mild or low residual MR and also reported a significant improvement in symptoms.

### Intrepid

The Intrepid (Medtronic, Minneapolis, MN, USA) is a dual stent design with the outer stent engaging the annulus, and the circular inner stent housing a 27 mm tricuspid bovine pericardial valve. Fixation is achieved by perimeter oversizing and facilitated by small cleats promoting frictional elements and tissue ingrowth. It is implanted transapically, it is retrievable and a transseptal delivery system is also under development.

Early experience data were described in the Intrepid Global Pilot Study, including a total of 50 patients mostly affected by functional MR (20). Technical success reached 98% with no device malfunction/thrombosis reported. The mortality rates were 14% and 24% after 30-day and 1-year follow-ups, respectively. After a 173-day median follow-up, MR grade was no more than mild in all patients who received implants and both symptoms as well as quality of life were improved. Recent evidence combining the data from the Pilot study and the APOLLO I was presented by Leon at TVT congress 2021: the all-cause mortality reached 38% at a 2-year follow-up while durable results were observed for the MR, with only 1% showing MR greater than or equal to moderate at a follow-up (21). Of note, an arm of the APOLLO trial (NCT03242642) will compare TMVI vs. TEER.

### Evoque

Evoque (Edwards Lifesciences, Irvine, CA, USA) consists of a trileaflet bovine pericardial valve mounted within a circular, self-expanding nitinol frame, covered with polyester to reduce PVL. Fixation is provided by the two opposing sets of anchors that capture the native leaflets. Both transfemoral and transapical delivery systems have been developed (22).

A total of 15 patients undergoing the transfemoral/transseptal approach have been enrolled so far, including compassionate use (*n* = 8) and early feasibility study (*n* = 7) cases (23). Technical success was 93% with 1 patient requiring a conversion to surgery. At a 30-day follow-up, 1 death (7%) was observed while the PVL closure and LVOTO requiring intervention (ASA) were needed in 2 patients and 1 patient, respectively.

### Highlife

The HighLife transcatheter MV replacement device (HighLife SAS, Paris, France) is a two-component system: first, a subannular implant (SAI) is deployed through a transfemoral retrograde transaortic route, then the valve prosthesis is implanted and anchored by interacting with the SAI. Current available experience is based upon 15 cases (24): a successful implantation was obtained in 13 patients while 2 cases required a conversion to surgery. The values of 30-day and 1-year mortality were 20 and 27%, respectively. No PVL was observed while only 1 case of LVOTO was reported.

### Sapien M3

Sapien M3 (Edwards Lifesciences, Irvine, CA, USA) is a balloon-expandable valve with a cobalt-chromium frame and three bovine leaflets and is implanted through the transfemoral/transseptal route. It is similar to the Sapien 3 valve with an additional skirt to reduce PVL risk.

Early results from the Early Feasibility Study on 35 high-risk patients showed 87% technical success and two cases where no valve was deployed (25). After a 30-day follow-up, one death was recorded and while, among the survivors, 88% showed MR grade ≤ 1+.

### Cardiovalve System

**Figure 2:**
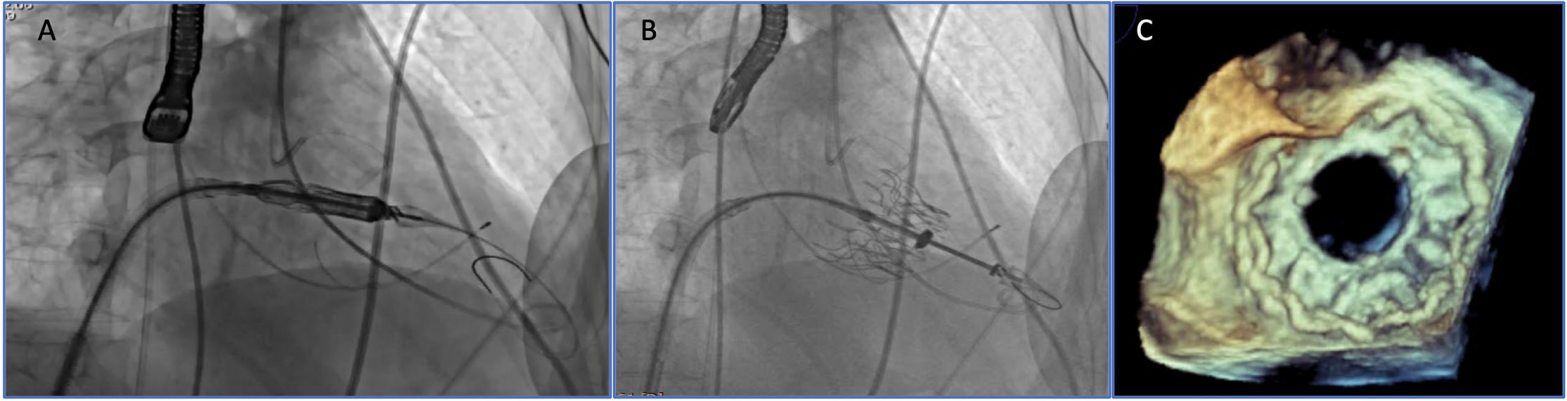
Transfemoral TMVI: cardiovalve device before **(A)** and after implantation **(B,C)**.

The Cardiovalve system (Valtech Cardio Ltd., Or Yehuda, Israel) features a self-expanding pericardial bovine valve mounted on a nitinol frame, specifically designed to be delivered through a transfemoral transseptal approach (Figure 2). The low device profile limits the risk of LVOTO while allowing tridimensional maneuvering within the left atrial and ventricular chamber, to achieve an optimal alignment to leaflet grasping.

Currently, a prospective, multicenter, and single-arm pilot study is underway (the AHEAD trial). Data about the first five patients showed a successful implantation in 100% of the cases with 60% of 30-day mortality, mainly related to access site complications (26).

### Other TMVI Devices

The Cephea system (Cephea Valve Technologies, Inc., San Jose, CA, USA) is a self-expanding trileaflet bovine pericardial valve. It is fully repositionable and recapturable and specifically designed for transatrial and transseptal delivery. First-in-human experience has been recently described (27): the valve was successfully implanted through a transseptal approach and an echocardiographic assessment after the valve implantation showed 2 mmHg transvalvular gradient and no regurgitation. After a 28-week follow-up, echocardiographic parameters were unchanged and the patient was in the NYHA I functional class.

Altavalve (4C Medical Technologies, USA) is a self-expanding, spherical shaped, nitinol, and three bovine leaflet device. It has a unique design as it self-anchors within the left atrium (LA) and without any active engagement with MV or left ventricle, abolishing the risk of LVOTO. Currently, only first-in-human case has been described (28). It is implanted through the transapical approach. Its procedure was technically successful and after a 30-day follow-up, no adverse events were reported. Clinical experience is expanding rapidly and an early feasibility study (NCT03997305) is underway.

## Repair or Replacement?

Currently, both American and European guidelines recommend the transcatheter edge-to-edge therapy as a possible treatment for primary, severely symptomatic, MR deemed at a high risk for surgery (class IIa) (3, 4). However, there is no mention of TMVI and how to select the best candidate for each treatment option.

According to surgical experience, MV repair should be considered as the first option treatment to address severe MR. Actually, there are a number of reasons to consider MV repair over MV replacement. First, TEER with MitraClip sets the bar high in terms of safety and efficacy: based on a recent systematic review comparing COAPT results (302 patients) with the pooled data of multiple TMVI devices (308 patients) the 30-day mortality value was 2.3 vs. 13.6% (12). Second, MV repair is more respectful of MV complex (leaflets, annulus, chordae, papillary muscles, LA, and the aortic valve continuity). Third, in spite of the Tendyne CE approval, the screening process is longer and more complex than the candidate selection for MitraClip, contributing to consider TMVI only for those cases where repair is unfeasible or challenging. Finally, there are several open issues for TMVI (see Section “Open issues”) limiting its use on a wider scale. Furthermore, the “one valve fits all” label for TMVI is only theoretical due to the high selection failure mainly related to the very strict anatomical eligibility criteria. On the other hand, TEER might be challenging in some MV anatomies with unfavorable features:

- Commissural/complex lesions.
- Multiple jets.
- Severe leaflet calcification.
- Baseline transvalvular gradient.
- Large coaptation gap.

In such anatomies, TMVI should be considered as a possible treatment. The main advantage for TMVI is its reproducibility with MR complete resolution in almost all cases whereas moderate/severe residual MR is more common in patients undergoing a repair with MitraClip (12). In addition, MV repair is less predictable and more operator-dependent as demonstrated by the recent data from the TVT registry showing how the experiences of institutions and operators impact MV repair outcomes (29).

Finally, another additional feature to consider in the choice of MR treatments is that TEER does not preclude TMVI thanks to the ELASTA-Clip technique (30): it consists of an intentional electrosurgical laceration of the anterior mitral leaflet (AML) leaving the clip fastened to the posterior leaflet allowing, in this way, TMVI also in those patients with prior TMVI with edge-to-edge clipping.

In this perspective, the roles of TEER and TMVI are likely to be more complementary rather than competitive although the best candidate for each treatment is still to be defined. Current studies on TMVI (e.g., Apollo trial, NCT03242642) have also introduced the transcatheter edge-to-edge arm to directly compare the two therapies and to define better the characteristics of those patients who may benefit the most from TEER or TMVI. Moreover, the data from the CHOICE MI trial (NCT04688190), comparing all available treatments for MR (TMVI, TEER, surgery, and medical therapy) in an observational and a retrospective fashion, will shed a new light on MR therapies, and will help to define the role of TMVI.

## Open Issues

Although LVOTO represents the most common limitation for TMVI, several issues need to be addressed in the decision process for severe MR treatment (Figure 4).

### Left Ventricle Outflow Tract Obstruction

It represents the most common limit in the screening process as well as the most common post-procedural complication, especially in the ViMAC subset of patients. Moreover, it is an independent predictor of 1-year mortality (31).

Currently, no univocal definition for LVOTO has been established. According to MVARC, iatrogenic LVOTO is defined by an increase in peak LVOT gradient >10 mmHg from baseline, as assessed by echocardiography. Alternatively, a peak gradient of >30 mm Hg, with hemodynamically significant LVOTO as a peak gradient >50 mm Hg has been used in some studies (32). Such different definitions might explain the different incidences of LVOTO among different studies. In general, all the three definitions might be applied, however, to standardize outcome definitions, it would be advisable to follow the MVARC definition.

A careful pre-procedural planning is of utmost importance with special attention to the possible anatomical risk factors for LVOTO (Table 3) (33). Among these, the neo-LVOT and the AML length/redundancy are highly predictive of LVOTO: although no univocal cut-off exists, a neo-LVOT >200 mm^2^ and an AML < 22 mm might enough to significantly reduce or to abolish the risk of LVOTO. However, it is worth to note that two more features may influence the risk of LVOTO:

- *Prosthesis profile*: a prosthesis with large profiles/height increases the risk of LVOTO as it tends to protrude in the left ventricle.
- *Implantation depth*: a deeper implantation increases the risk of LVOTO. However, it should be balanced with the risk of thrombosis for too high/atrial implantation.

**Table 3:** Risk factor for LVOT obstruction.

**Anatomical**	
Predicted neo-LVOT (CT scan)	<170–190 mm^2^
Basal septal hypertrophy	>15 mm
Aorto-mitral angle	<130°
AML length/redundancy	>22 mm
Small ventricle (MA-to-IVS)	<18 mm
**Device/procedural**	
Large profile	
Deep/ventricular implant	

To address such a complication, a fully percutaneous technique aiming at intentional laceration of the AML has been described and successfully applied in 30 patients who are at a high risk for LVOTO (LAMPOON IDE, NCT03015194) (34, 35). However, the LAMPOON technique is technically demanding and requires highly expert operators, whereas larger data with a longer follow-up are needed.

An alternative solution is represented by transcoronary ASA. However, if ASA allows a significant increase in the LVOT area, on the other hand, it might be a risk for an injury to the conduction system. In addition, myocardial infarction is a direct consequence of the procedure, and left ventricle remodeling is usually obtained after 2–4 weeks (36).

### Sealing and Fixation

Current available devices are based on different fixation mechanisms: apical tethering (e.g., Tendyne), native leaflet engagement (e.g., Tiara), mitral annulus clamping (e.g., Evoque), and radial force (e.g., Intrepid). However, several anatomical issues complicate prosthesis fixation in the mitral position: the asymmetrical shape of the mitral annulus and leaflets, large annular dimensions, the absence of calcifications in most cases, and the complex subvalvular anatomy. Currently, there is no evidence on the efficacy of different fixation mechanisms while valve embolization represents a threatening complication occurring in up to 7% of patients in the ViMAC subgroup (8). Alternatively, inadequate sealing might cause PVL that has been observed in up to 8% of patients undergoing ViR (8). In this perspective, D-shaped design (e.g., Tendyne and Tiara) might adapt better to MV annulus and decrease the risk for leakage.

### Anticoagulation

**Figure 3:**
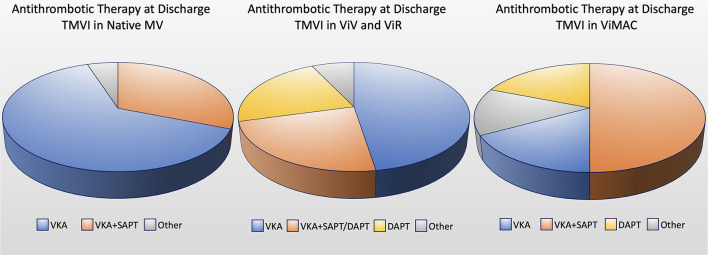
Circle chart showing the anticoagulant/antiplatelet therapy at discharge for TMVI in native mitral valve, valve-in-valve (ViV), valve-in-ring (ViR) and valve-in-mitral annular calcification (ViMAC) [data based on Ref. (33)]. DAPT, double antiplatelet therapy; SAPT, single antiplatelet therapy; VKA, vitamin K antagonist.

Although vitamin-K antagonist is the most used drug, almost all molecules from antiplatelet to anticoagulants drugs, also in different combinations, have been used so far. However, currently, no univocal approach has been established for a long-term drug regimen in patients undergoing TMVI. Multiple factors may influence the risk for thrombosis: patient-, prosthesis- (e.g., leaflet or stent material), and procedure-related factors (high implantations in LA increase the thrombogenic risk) (37). In general, long-term antiplatelet/anticoagulant regimens are advisable and might represent an issue to be considered in the MV therapy selection, especially in young or low-risk categories (Figure 3).

**Figure 4:**
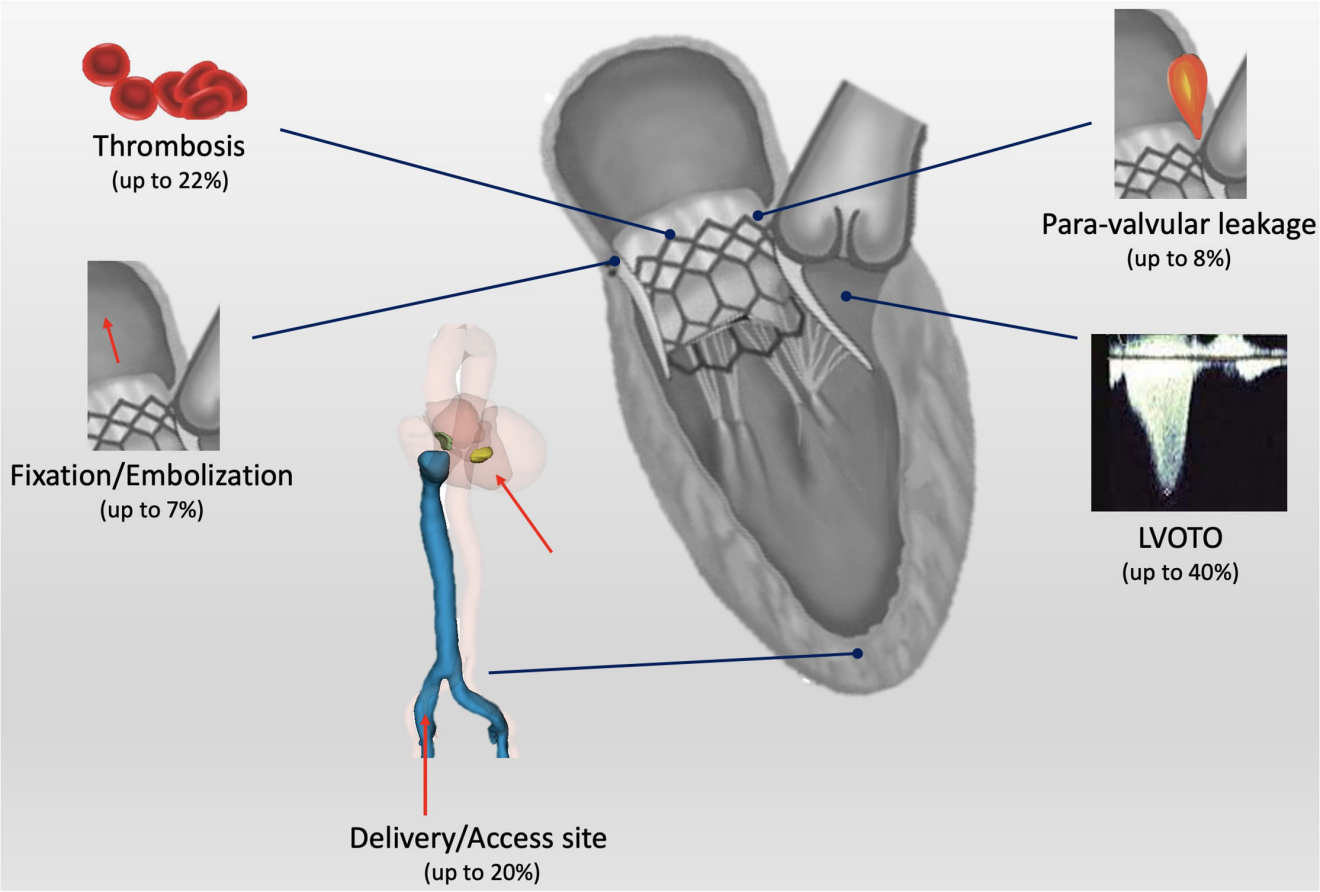
Central illustration: current open issues for transcatheter mitral valve implantation (TMVI). LVOTO, left ventricle outflow tract obstruction.

### Durability

Long-term durability represents an open issue for prostheses in both mitral and aortic positions. The lesson learned from the surgical bioprosthesis is that the degeneration process begins at 5 years after MV intervention, and the freedom from a structural valve deterioration varies from 70 to 90% at 10 years, with mitral position bioprosthesis deteriorating earlier than the aortic ones (38). Current experience with TMVI is still at its early stages, and the follow-up data are still limited to draw conclusions about long-term durability.

### Delivery

Most of the current experience is based on the transapical approach although apical tear, life-threatening apical bleeding, myocardial damage, coronary damage, and infections might complicate this approach.

The transfemoral/transseptal route might be more appealing although it is technically demanding as a precise and safe transseptal puncture is required (39). Prosthesis coaxility and maneuverability represent another technical limitation for the transseptal access. Moreover, from an engineering standpoint, two main challenges should be overcome: reduced delivery caliber with adequate flexibility to allow the maneuverability and trackability of the device for precise positioning. In spite of the large bore delivery sheaths, according to current evidences, an atrial septal defect closure is needed in <10% of cases and the main indications for closure are the right-to-left atrial shunt or bidirectional shunt.

## Future Directions

Transcatheter MV repair shows a wider experience and remains a mainstay in the percutaneous treatment of MR due to multiple reasons:

- It is versatile and, in expert hands, might be used for complex anatomies and for different etiologies.
- It is safe and effective (3).
- It is a relatively simple procedure (40).
- The available follow-up and the overall experience are wider as compared to TMVI.

Transcatheter MV implantation field has still some limitations to overcome although much experience and several data have been collected in the last few years. LVOTO represents an important limitation for TMVI in all subsets, with special regards to ViMAC. The LAMPOON technique can be a possible solution to LVOTO although it requires highly experienced operators and might be not applicable to all cases. Larger data with a longer follow-up are needed to better understand the best candidate and right timing for TMVI procedures.

In the setting of ViV, ViR, and ViMAC, the role of TMVI is much clearer: given the very promising results, it might play a primary role in the setting of ViV (41). Encouraging mid-term clinical and hemodynamic outcomes for ViV has been documented in a single-center experience that has also demonstrated how surgical valve size could have an effect on hemodynamic results: the larger the surgical valve size, the better the hemodynamics (42). Every effort to implant relatively oversized surgical valves, especially in young patients, should be done to allow a safe and an effective TMVI in the future, especially related to suboptimal hemodynamic performance. In the ViR scenario, further data are needed to better understand the type of rings in which the prosthetic valve might adapt the best. Nowadays, to facilitate the approach to ViV and ViR, operators can also rely on a smartphone app, which easily provides details of surgical valves/ring designs and their compatibility with currently available transcatheter valves, allowing a safe planning of the VIV or VIR procedure (43).

Finally, ViMAC is the category with the worst outcomes: a careful patient selection and Heart Team multidisciplinary assessment might help to understand those who may benefit from TMVI.

A few studies comparing TMVI with TEER will provide fundamental information and will indicate the best candidate for each treatment. Based on the current available data, TMVI is going to enrich the MV therapy toolbox and to play a complementary role with TEER. Heart Team, and more specifically Heart Valve Clinics, plays a major role in the treatment choice taking into account procedure safety and efficacy according to MR etiology and anatomy, timing, and the experience of institutions/operators.

## Conclusions

Transcatheter MV implantation has grown slowly over the last few years as compared to TEER. However, the recent approval of the Tendyne device along with some technical improvements (e.g., LAMPOON and ELASTA-Clip) has increased the attention on this complex field. Although TEER with MitraClip represents the main percutaneous treatment and has been introduced in the latest American and European guidelines as a treatment option, TMVI will widen the available MV therapies and might be indicated in those cases where both surgery and TEER are contraindicated or highly challenging (3, 4). Despite being appealed, the “one device fits all” myth is still far from becoming true due to the multiple open issues related to TMVI world. Ongoing studies will help to understand strengths and possible limitations on the role for TMVI among the available transcatheter MV therapies.

## Author Contributions

GR and FM contributed to manuscript conceiving and revision. GM, GT, EMo, MB, AM, and AB contributed to data collection and literature review. GC, EMi, and GS provided manuscript final supervision. All authors contributed to the article and approved the submitted version.

## Conflict of Interest

FM is a consultant for Abbott Vascular, Medtronic, Edwards Lifesciences, Perifect, Xeltis, Transseptal Solutions, Magenta and Cardiovalve, has received grant support from Abbott Vascular, Medtronic, Edwards Lifesciences, Biotronik, and Boston Scientific, NVT, Terumo, has received royalties from Edwards Lifesciences and 4Tech, and is co-founder/shareholder of Transseptal Solutions, 4Tech, Cardiovalve, Magenta, Perifect; Coregard and SwissVortex. The remaining authors declare that the research was conducted in the absence of any commercial or financial relationships that could be construed as a potential conflict of interest.

## Publisher's Note

All claims expressed in this article are solely those of the authors and do not necessarily represent those of their affiliated organizations, or those of the publisher, the editors and the reviewers. Any product that may be evaluated in this article, or claim that may be made by its manufacturer, is not guaranteed or endorsed by the publisher.
